# Prolactin-Releasing Peptide System as a Potential Mechanism of Stress Coping: Studies in Male Rats

**DOI:** 10.3390/ijms26094155

**Published:** 2025-04-27

**Authors:** Evelin Szabó, Viktória Kormos, Zsuzsanna E. Tóth, Dóra Zelena, Anita Kovács

**Affiliations:** 1 Institute of Physiology, Medical School, University of Pécs, Centre for Neuroscience, Szentágothai Research Centre, H7624 Pécs, Hungary; szabo.evelin2@pte.hu (E.S.); anita.kovacs@aok.pte.hu (A.K.); 2 Department of Pharmacology and Pharmacotherapy, Medical School, University of Pécs, H7624 Pécs, Hungary; viktoria.kormos@aok.pte.hu; 3 Department of Anatomy, Histology and Embryology, Semmelweis University, H1094 Budapest, Hungary; toth.zsuzsanna.emese@semmelweis.hu

**Keywords:** prolactin-releasing peptide, depression, forced swim test, active coping, passive coping, mRNA expression, RNAscope

## Abstract

Prolactin-releasing peptide (PrRP) has a regulatory role in both acute and chronic stress, suggesting its potential contribution to stress-related disorders such as depression. However, not all individuals with depression respond equally to stressors. We aimed to determine whether the PrRP system could underlie stress coping, an important aspect of depression. The forced swim test was used both as a stressor and as a method to assess coping strategy. Based on immobility time, active coping and passive coping subgroups were identified, and 10 brain regions were studied using qPCR to measure the mRNA expression levels of *PrRP* and its receptors (specific: *GPR10*; non-specific: *NPFFR2*). Passive coping animals spent more time in an immobile posture and exhibited altered mRNA expression levels in the medullary A1 region, the habenula, and the arcuate nucleus than control or active coping rats. Additionally, we identified corticotropin-releasing hormone and vesicular glutamate transporter 2 positive neurons in the A1 medullary region that contained *Prrp*, suggesting a modulatory role of PrRP in these excitatory neurons involved in stress regulation. Our findings reinforce the hypothesis that PrRP plays a role in stress coping, a process closely linked to depression. However its effect is brain region-specific.

## 1. Introduction

With increasing life expectancy, mental health and quality of life are becoming increasingly important. Stress-related disorders, like anxiety and depression, have a devastating impact on individuals, families, and society [1]. The COVID-19 pandemic has further worsened mental health outcomes [2,3,4,5]. By 2030, the World Health Organization predicts that depression, which very often involves a passive coping strategy, like avoidance or rumination [6,7,8], will be the leading cause of disability-adjusted life years lost [9]. Understanding its molecular mechanism is crucial for developing effective, personalized treatments, as current therapies, including selective serotonin reuptake inhibitors and serotonin and norepinephrine reuptake inhibitors, fail in about 30% of patients. Even augmentation (e.g., lithium, T3, antipsychotics) or drug repurposing (e.g., ketamine, cyclooxygenase 2 inhibitors, psilocybin, infliximab) is still ineffective in one-third of the treatment-resistant patients [10], highlighting the need for new therapeutic targets.

Prolactin-releasing peptide (PrRP), a member of the RFamide neuropeptide family, has recently gained attention for its role in stress and related pathologies [11]. Its involvement in depression is particularly relevant, as altered PrRP mRNA expression has been observed in the brains of suicidal individuals [12]. Indeed, PrRP plays a key role in regulating acute and chronic stress [13,14], making it significant in stress-related psychopathologies. Primarily expressed in the noradrenergic A1 and A2 cell groups of the medulla oblongata (in co-expression with noradrenaline) [15,16], PrRP transmits stress-related information from the brainstem to the hypothalamus, including the paraventricular nucleus (PVN), the center of the hypothalamic–pituitary–adrenocortical (HPA) axis [17]. Lower *PrRP* expression is also found in the dorsomedial nucleus of the hypothalamus (DMN) in non-noradrenergic neurons [12]. PrRP fibers primarily target the hypothalamus, particularly the PVN, lateral hypothalamus, and DMN [12,18]. PrRP binds to the GPR10 receptor [19,20] but also acts as an agonist for the neuropeptide FF receptor 2 (NPFFR2) [21], both of which are widely distributed in brain regions associated with depression, such as the PVN [22] and amygdala [23]. Intracerebroventricular PrRP administration activated the HPA axis, leading to increased plasma adrenocorticotropin (ACTH) and corticosterone levels via the PVN [24,25,26,27]. It also raised arterial blood pressure, reflecting sympathetic activation [24,25,26,27]. Chronic stress, such as repeated restraint, increased *Prrp* mRNA expression in the brainstem, shifting the PrRP/tyrosine hydroxylase (TH) ratio in favor of PrRP [13]. This suggests a crucial role for PrRP in adapting to chronic stress, potentially protecting against the mental health consequences of prolonged stress [28].

Here, we investigated changes in PrRP and its receptors at the mRNA level in a preclinical model widely used to assess passive coping strategies closely associated with depression. Given individual differences in stress susceptibility, which may impact treatment strategies, we compared active coping (A) and passive coping (P) animals. Although depressive-like symptoms can be studied in animals using many methods (see Supplementary Table S1), we chose the forced swim test (FST, Porsolt test), validated for both rats and mice [29,30]. Despite intensive debate [31,32,33,34], FST has some validity in predicting a compound’s antidepressant potential. The major criticism is that it measures coping strategy rather than a depressive-like state. However, the passive coping observed in FST was linked to depression in many studies [6,7,8]. Moreover, FST is a short, not severe stress [35], it is easy to conduct, and active and passive coping animals can be easily separated [36]. To minimize variability from female cyclic changes [37] and sex-dependent stress-induced PrRP alterations [13], only male rats were included. Medullary PrRP-positive cells in the A1 region were analyzed using immunohistochemistry (protein detection) combined with RNAscope in situ hybridization (mRNA detection).

## 2. Results

### 2.1. FST Increased Passive Coping in Vulnerable Animals

**Figure 1:**
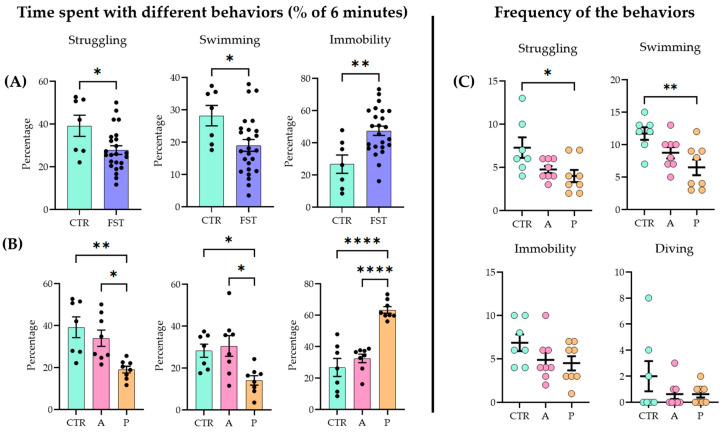
Behavioral outcomes in the forced swim test (FST) (**A**) One day after a 15 min forced swim session, the animals exhibited an increased passive coping strategy compared to naïve controls (CTRs) during a 6 min test. (**B**) When the FST group was divided further into active coping (A) and passive coping (P) subgroups based on immobility time, the A group showed no significant difference from the controls (CTRs), while the P group differed from both the CTR and A groups. (**C**) Active coping animals displayed active behaviors (struggling and swimming) more frequently than the other groups. Data are expressed as mean ± SEM. Dots represent individual data points. * *p* < 0.05, ** *p* < 0.01, and **** *p* < 0.0001 vs. the labeled group.

When naïve and previously stressed (FST 24 h earlier) animals were subjected to a 6 min FST, all the main parameters (struggling, swimming, and immobilization) showed significant differences between the two groups (unpaired two-tailed *t*-test: struggling *p =* 0.019, t = 4.488, df = 29; swimming *p* = 0.0222, t = 2.411, df = 30; immobility *p* = 0.002, t = 3.327, df = 29; control (CTR) n = 7; FST n = 24) (Figure 1A; for raw data, see Supplementary Table S2; for detailed statistics, see Supplementary Table S3).

Active (A) and passive (P) coping subgroups were separated based on the behavioral data (see Section 4.2). Upon analysis (ANOVA and Tukey’s post hoc test), the A group did not differ significantly from the controls (CTRs), while both the CTR and the A groups showed significant differences from the P group (struggling F(2, 20) = 8.354, *p* = 0.002; swimming F(2, 20) = 6.069, *p* = 0.0087; immobility F(2, 20) = 29.51, *p* < 0.0001) (Figure 1B). In terms of frequency (how many times the animal started the behavior), P animals began the two active behaviors fewer times than the other two groups (struggling F(2, 20) = 4.488, *p* = 0.0245; swimming F(2, 20) = 6.165, *p* = 0.0082). There were no differences among groups in immobility (F(2, 20) = 1.195, *p* = 0.1622) and diving frequencies (F(2, 20) = 1.359, *p* = 0.2797) (Figure 1C).

### 2.2. A1, Arcuate Nucleus, and Habenula Showed mRNA Expression Differences in the PrRP System Parallel with FST Sensitivity

**Figure 2:**
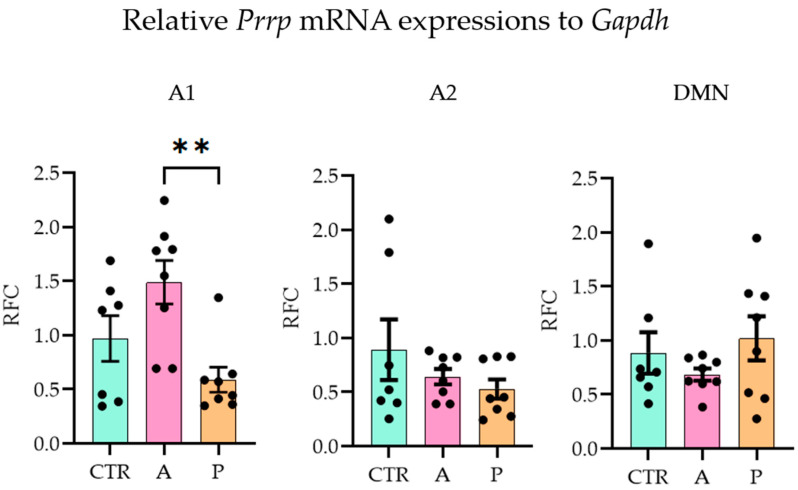
Relative Prrp mRNA expression level changes in the three Prrp-producing brain regions. Active coping animals displayed a higher expression of *Prrp* than passive coping animals in the medullary A1 region. Abbreviations: A1 and A2—medullary noradrenergic nuclei, A—active coping group, CTR—control group, DMN—dorsomedial nucleus of the hypothalamus, *Gapdh*—glyceraldehyde 3-phosphate dehydrogenase, P—passive coping group, RFC—relative fold change. Data are expressed as mean ± SEM. Dots represent individual data points. ** *p* < 0.01, vs. the labeled group.

With the quantitative polymerase chain reaction (qPCR), we examined the three major sites of PrRP expression (A1, A2 (part of the nucleus tractus solitarius, NTS [38]), DMN [39]) and ten possible target brain areas (receptor expressions, see Section 4.3) on the mRNA level (one-way ANOVA with Tukey’s multiple comparison) (threshold cycle (CT) values are available in Supplementary Table S4). In A animals, *Prrp* expression in the A1 region was significantly higher than in P group (ANOVA F(2,20) = 6.705, *p* = 0.006; Tukey’s multiple comparison *p* = 0.004 between A and P), while the CTR and P groups did not differ from each other, indicating an inadequate response of the P animals to the repeated stress. The other two brain areas, A2 and DMN, did not show group differences (Figure 2).

**Figure 3:**
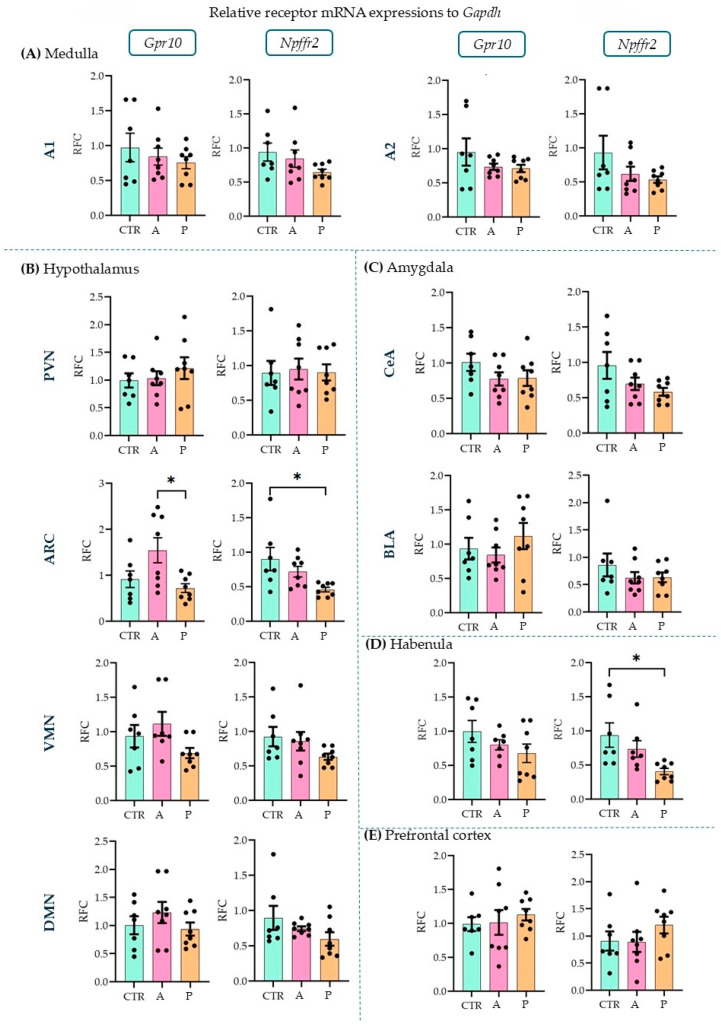
Relative mRNA expression changes in the Gpr10 and Npffr2 levels. A higher Gpr10 expression was found in the arcuate nucleus in active compared to control and passive coping animals. Passive coping animals displayed a lower expression of Npffr2 than controls in both the arcuate nucleus and habenula. mRNA abbreviations: Gpr10—specific receptor to PrRP and Npff2—neuropeptide FF receptor 2; groups: CTR—control, A—active coping, and P—passive coping; brain regions: A1 and A2—noradrenergic nuclei of the medulla, VMN—ventromedial nucleus of the hypothalamus, DMN—dorsomedial nucleus of the hypothalamus, PVN—paraventricular nucleus of the hypothalamus, ARC—arcuate nucleus, CEA—central amygdala, BLA—basolateral amygdala, HAB—habenula, and PFC—prefrontal cortex. Data are expressed as mean ± SEM. Dots represent individual data points. * *p* < 0.05, vs. the labeled group.

Upon investigating the receptors, in the ARC (Figure 3B), we detected a higher expression level of *Gpr10* in P than in A animals (ANOVA F(2, 20) = 4.856, *p* = 0.019; Tukey’s multiple comparison *p* = 0.019 between A and P). P animals, however, had lower *Npffr2* expression levels here than CTRs (ANOVA F(2, 20) = 4.736, *p* = 0.021; Tukey’s multiple comparison *p* = 0.017 between CTR and P) and the same difference was found in the habenula (HAB, Figure 3D) (ANOVA F(2, 19) = 5.001, *p* = 0.018; Tukey’s multiple comparison *p* = 0.015 between CTR and P). This suggests that PrRP signaling may be insufficient in these areas in response to repeated stressors. However, the other sevenbrain areas did not show alterations between the groups (Figure 3A–E). The relative fold changes and ANOVA results for all the regions are summarized in Table 1. The expression of both receptors has been previously described in the medulla, hypothalamus, and amygdala [11] but not in the HAB or prefrontal cortex (PFC). Here, we report the detectable mRNA expression levels of the receptors in both areas (Table 2).

**Figure 4:**
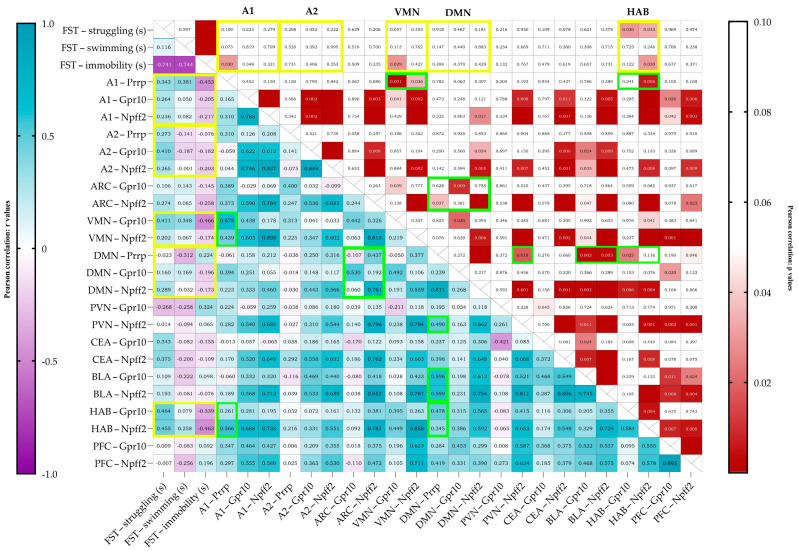
Pearson’s correlation data visualized on a heatmap with r values on the left and *p* values on the right. On the left, a strong positive correlation is shown in darker shades of blue, and a strong negative correlation is shown in purple. Boxes are labeled with an r value of up to 3 decimals. On the right, cells with values over 0.05 are left blank (white), and under 0.05, the lower the *p* value, the darker the shade of red shown. The cells are labeled with the exact p values, and if the cell is empty, the p value is below 0.001. FST data are given in seconds (FST—forced swim test). Yellow frames represent the important correlations between behavior and mRNA expression, and green frames represent the noteworthy correlations between *Prrp* production and receptor expression. mRNA abbreviations: Prrp—prolactin-releasing peptide, Gpr10—specific receptor to PrRP, and Npff2—neuropeptide FF receptor 2; brain regions: A1 and A2—noradrenergic nuclei of the medulla, VMN—ventromedial nucleus of the hypothalamus, DMN—dorsomedial nucleus of the hypothalamus, PVN—paraventricular nucleus of the hypothalamus, ARC—arcuate nucleus, CEA—central amygdala, BLA—basolateral amygdala, HAB—habenula, and PFC—prefrontal cortex.

Upon correlating the results of the behavioral test and the mRNA expressions (Pearson correlation), immobility negatively correlated with *Prrp* production in the A1 region (r = −0.453, *p* = 0.030) (Figure 4). The struggling behavior of the animals positively correlated with the relative expression of both receptors’ mRNA in the HAB (*Gpr10* r = 0.464, *p* = 0.030, *Npff2r* r = 0.455, *p* = 0.033). Immobility correlated negatively with the receptor expressions in the HAB; however, only with *Npff2r* did it reach significance (*Gpr10* r = −0.339, *p* = 0.122, *Npff2r* r = −0.463, *p* = 0.030). Immobility also correlated negatively with the *Gpr10* expression in the VMN (r = −0.466, *p* = 0.029).

There were also several positive correlations between PrRP production and receptor expressions. *Prrp* mRNA levels in the medullary A1 region correlated with the receptor levels in the VMN, showing a moderate correlation with *Gpr10* (r = 0.675, *p* = 0.001) and a low correlation with *Npff2r* (r = 0.439, *p* = 0.036). A1 *Prrp* levels showed a moderate correlation with *Npff2* (r = 0.566, *p* = 0.006) levels in the HAB. *Prrp* mRNA expression in the medullary A2 region did not correlate with any other site’s *Prrp* nor with any receptor *mRNA* expressions. *Prrp* mRNA expression levels and *Npff2r* expression in the DMN showed a high correlation (r = 0.811, *p* < 0.0001). DMN’s *Prrp* expression showed low-to-moderate correlation to *Npff2r* levels both in the ARC and the PVN (ARC *Npffr2* r = 0.437, *p* = 0.037; PVN *Npff2r* r = 0.490, r = 0.018). Interestingly, *Prrp* expression in the DMN showed moderate positive correlation to all receptor expression levels in the BLA (*Gpr10* r = 0.596, *p* = 0.003; *Npff2* r = 0.599, *p* = 0.003) but not in the CEA (*Gpr10* r = 0.237, *p* = 0.276; *Npff2* r = 0.389, *p* = 0.059). Positive correlation with *Prrp* production in DMN was also found in the HAB’s and PFC’s receptor expressions; specifically, in the HAB, the *Gpr10,* while in the PFC, the *Npff2r* expression was significant (*Gpr10* in HAB r = 0.478, *p* = 0.025; *Npff2r* in PFC r = 0.419, *p* = 0.046).

### 2.3. In Situ Hybridization Reveals New Characteristics of Medullary A1 Cells

**Figure 5:**
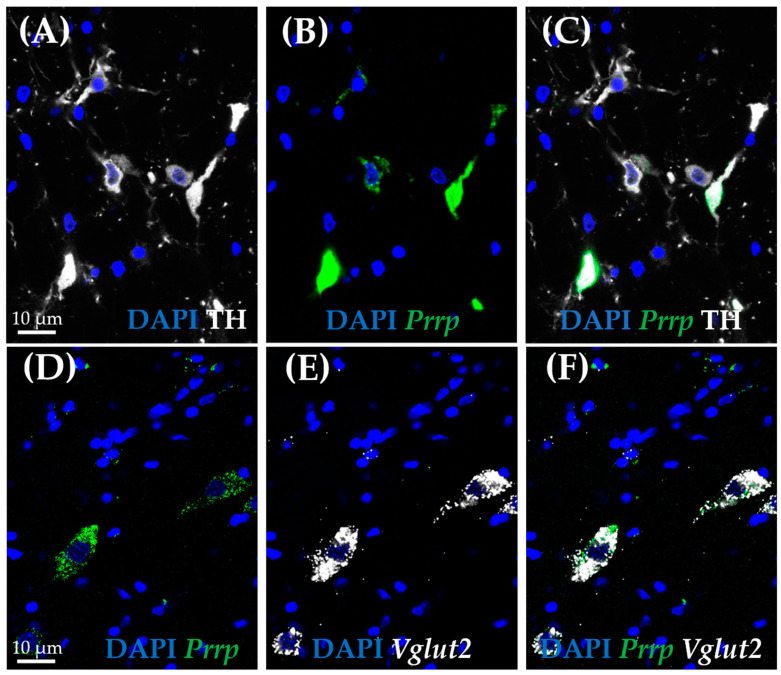
Co-expression analysis of the prolactin-releasing peptide-positive neurons in the A1 cell group by RNAscope in situ hybridization combined with immunostaining. Representative fluorescence images showing the co-localization of prolactin-releasing peptide mRNA (**B**,**D**; *Prrp*, green) with vesicular glutamate transporter 2 mRNA (**E**,**F**; *Vglut2*, white), as well as tyrosine hydroxylase (**A**,**C**; TH, white). Nuclei were stained with 4′,6-diamidino-2-phenylindole (DAPI, blue).

**Figure 6:**
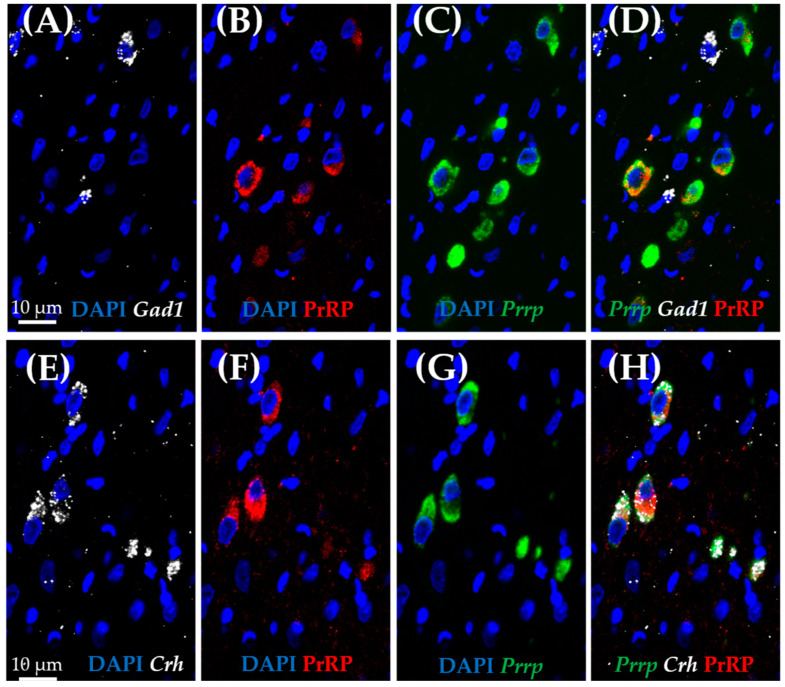
Characterization of prolactin-releasing peptide-positive neurons in the medullary A1 region by RNAscope in situ hybridization combined with immunostaining. Representative fluorescence images showing the co-localization of prolactin-releasing peptide (**B**,**F**; PrRP, red) and mRNA (**C**,**G**; *Prrp*, green) with corticotropin-releasing hormone mRNA (**E**,**H**; *Crh*, white). Note that there is no co-localization with glutamate decarboxylase 1 mRNA (**A**,**D**; *Gad1*, white). Nuclei were stained with 4′,6-diamidino-2-phenylindole (DAPI, blue).

As medullary A1 neurons were deeply implicated in active coping, resembling resiliency to depression-like behavior, we used the RNAscope in situ hybridization (ISH) technique to characterize these neurons. In concordance with the literature, *Prrp* mRNA-containing neurons can be found in this region, and they proved to be noradrenergic, staining positive with tyrosine–hydroxylase antibody (TH) (Figure 5A–C). More precisely, 85.71% of TH-positive neurones were also positive for *Prrp* (Supplementary Table S6). To further characterize these cells, we hybridized vesicular glutamate transporter 2 (*Vglut2*) (Figure 5D–F) or glutamate decarboxylase (*Gad1*) mRNA probes alongside the *Prrp* probe and PrRP antibody (Figure 6A–D). As expected, the PrRP proteins were found in 100% of *Prrp* mRNA-positive cells. The mRNA copy number was very high, forming a confluent mRNA signal in many cases (e.g., Figure 5B, green). The positivity for *Vglut2* labels glutamatergic, excitatory neurons, while *Gad1* positivity labels GABA-ergic (γ-aminobutyric acid), inhibitory neurons. We found *Prrp* mRNA co-expression in *Vglut2*-positive (72% of *Vglut2*-positive cells) but not *Gad1*-positive (0%,) cells in the A1 area (Supplementary Table S6). Additionally, we examined the well-known stress hormone, corticotropin-releasing hormone (*Crh*) mRNA, which also showed co-localization with the *Prrp* probe (100%, Supplementary Table S6) as well as with the PrRP antibody (Figure 6E–H).

## 3. Discussion

We confirmed that behavioral passive coping in the FST model was accompanied by lower *Prrp* mRNA levels in the medullary A1 region, as well as lower specific *Gpr10* receptor levels in the ARC and lower *Npffr2* mRNA (non-specific receptor) concentration both in the HAB and the ARC. In accordance with the literature, the RNAscope *ISH* in naïve animals confirmed *Prrp* expression in the noradrenergic neurons of the medullary A1 region. Moreover, the *Prrp* mRNA as well as protein signal was present in *Vglut2-* and *Crh*-positive A1 medullary neurons, highlighting PrRP’s role in neuronal excitation, which might be especially important during stress.

PrRP has a role in regulating the HPA axis during both acute and chronic stress [13,40,41], and our previous results also suggested its role in depression using human samples [12]. In our FST model, out of the three main PrRP-producing brain regions (A1, A2/NTS, DMN), only the medullary A1 region showed different *Prrp* mRNA expression in A than P individuals. Moreover, the immobility time negatively correlated with the A1 *Prrp* mRNA levels, suggesting that an increased passive coping strategy is accompanied by lower *Prrp*. As all animals underwent the 6 min FST section, we can exclude swimming-induced changes. Thus, the observed *Prrp* mRNA difference might be a possible determinant of the stress vulnerability of the rats. This is in line with our previous findings on the importance of the A1 region in stress adaptation [13,14]. Moreover, high *Prrp* mRNA content was also detected in the A1 region of P rats using another depression model, the learned helplessness [12]. However, in that case, the *Prrp* difference in the DMN also underlined the vulnerability. The present FST is a short intervention, while the learned helplessness procedure is more severe (electric foot shock for 40 min) and prolonged (tested 7 days after training). We might assume that regulating the A1, as one of the centers of the sympathetic system [42], is more important during short interventions, while the role of DMN might come into focus during chronic situations [43]. However, it is clear that DMN has an important regulatory role during an acute stress situation as well, e.g., by increasing cardiovascular sympathetic activity [44] or regulating respiration [45]. Previously, the DMN PrRP neurons were implicated in feeding [46]. We might assume that learned helplessness-induced anhedonia [47] influenced food intake, thereby having a stronger impact on DMN than on A1. Although there was no significant difference in the *Prrp* mRNA level between P and A individuals, the DMN-PrRP levels positively correlated with the receptor levels on many stress-related areas like PVN and BLA as well as HAB and PFC. These correlations support that DMN-PrRP might form stress adaptation, as we have seen in the learned helplessness model [12]. However, it was also suggested that *Prrp* expression correlates with stress rather than with the individual’s vulnerability to develop depression [11].

We further characterized the medullary A1 neurons as a major PrRP-dependent determinant of stress vulnerability in FST. We found that these TH-positive noradrenergic neurons, besides containing *Prrp* mRNA and PrRP peptide [14], were also positive for *Vglut2* and *Crh* mRNA. Interestingly, PrRP receptors share similarity to AMPA (α-amino-3-hydroxy-5-methyl-4-isoxazolepropionic acid, a glutamate receptor) suggesting their interaction in signalization [48]. It is well known that the noradrenergic A1 cells [49,50] project to the PVN, the center of the HPA axis, where they might regulate CRH synthesis and release. Interestingly, CRH production was found in catecholaminergic brainstem nuclei most probably transmitting systemic stressor information (e.g., blood loss, pain, hypoglycemia) to the PVN [51]. Although, in the PVN, CRH can be found in excitatory VGluT2-positive cells, but in other brain areas, e.g., in the central amygdala (CeA) and bed nucleus of the stria terminalis (BNST), it is produced in GABAergic neurons [52]. Here, we confirmed its presence in excitatory *Vglut2-positive* A1 neurons, which makes these brainstem CRH cells more similar to the PVN-CRH neurons. We might assume that together with noradrenaline and PrRP, glutamate and CRH will also be transported to the PVN and participate in the fine-tuning of the stress response. In line with this assumption, the DMN *Vglut2*=positive neurons (which might contain PrRP as well) were previously confirmed to regulate CRH release [53]. Thus, we hypothesize that the noradrenergic PrRP-expressing neurons in the medullary A1 region through PVN innervation will influence glucocorticoid production, thereby coping with the stressors. Indeed, glucocorticoid elevation has been described to have a positive correlation with the vulnerable phenotype [54,55]. However, we cannot dismiss the possibility that the noradrenergic innervation of the CRHergic BNST is responsible for the different stress coping, as this brain area might be responsible for behavioral alterations as well as for transmitting information to the PVN [56]. In supporting studies, it has been previously shown that GPR10 is co-expressed extensively with CRH in the BNST [57].

In addition to these HPA axis effects, the cortico-habenular circuit has also been suggested as an important modifier of the stress response [58,59,60]. HAB dysfunction has been implicated in the pathomechanism of depression [61] and in vulnerability to stress [62]. Our passive coping rats showed reduced PrRP receptor signaling in this brain area, highlighting this circuit’s role in stress regulation. As additional support, *Npff2r* signaling in the HAB negatively correlated with immobility and positively with struggling time, and there was a positive correlation between A1 *Prrp* and HAB *Npffr2* expression as well. In mice, a GABA-ergic (inhibitory) signal from the lateral septum increased immobility in the tail suspension test through a parallel influence on HAB and DMN [63]. The positive correlation between HAB *Gpr10* receptor mRNA and DMN *Prrp* mRNA expression in our hands suggests that PrRP might be an important coordinator of stress coping.

In the ARC, both the specific and non-specific *Prrp receptor* mRNA levels were lower in passive coping individuals than in controls, suggesting lower PrRP signaling in this brain area. Silencing or overproducing interleukin 11 in the proopiomelanocortin (POMC)-positive cells of the ARC influenced depressive-like behavior in a mouse chronic unpredictable stress model [64]. The chronic chemogenetic stimulation of these POMC-ARC cells led to anhedonia and behavioral despair (i.e., increased immobility in FST), however, only in male but not female mice [65]. This suggests that POMC-ARC is a key hub regulating depression, including hypophagia and anhedonia [66]. PrRP might regulate the gonadotropin-releasing hormone secretion [67], which might contribute to sexual problems in depression, although this was linked more to the preoptic hypothalamic area than to ARC. However, the PrRP innervation of dopaminergic ARC neurons might regulate prolactin release and, in this way, influence sexual functions [68].

Interestingly, the specific PrRP receptor *Gpr10* expression in the VMN negatively correlated with immobility time and showed a positive correlation with A1 PrRP expression, similarly to HAB, however, without significant direct differences between the R and V groups. VMN is also a known satiety center [69]; thus, its role in stress adaptation might be related to energy homeostasis.

Our results strengthen the theory about PrRP’s role in stress adaptation, emphasizing its brain region specificity, with the most important role of medullary A1 cells. We confirmed the possible utility of the PrRP system as a therapeutic target in depression.

## 4. Materials and Methods

### 4.1. Animals

Male Wistar rats from the local colony were used (University of Pécs, Institute of Physiology, facility license number: BAHU0140L 17). All animals were housed individually, and tap water and standard chow were available ad libitum. The facility maintained a 12/12 dark–light cycle, experiments were conducted between 10 am and 3 pm during the animals’ dark/active period. For FST and quantitative PCR, 8-week-old adolescent rats (250–350 g) were used, while for the RNAscope, we used 6-month-old adult rats (450–500 g, n = 2). For qPCR, the brains were harvested by decapitation 30 min after the 6 min swimming session. The brains were snap-frozen on dry ice and stored at −80 °C. Animals for RNAscope were deeply anesthetized with 26% urethane (0.5 mL/100 g in body weight). The depth of anesthesia was determined by the diminished corneal reflex. These animals were perfused transcardially (with 0.1 M, pH = 7.4 PBS and 4% paraformaldehyde solution in the same PBS).

### 4.2. Forced Swim Test (FST)

First day (training): From the 31 animals, 7 were randomly selected and omitted this day (control group). The other 24 animals completed a 15 min forced swim session. We examined 3 animals at the same time in separate glass cylinders (high: 61 cm, diameter: 18 cm, filled up to 30 cm with tap water 24 ± 1 °C). The sessions were recorded with a digital camera.

Second day: All 31 animals completed a 6 min forced swimming session 24 h ± 20 min after their first swimming (protocol has been adjusted to include the controls). The session was recorded by a digital camera and later analyzed manually with the open access SolomonCoder behavioral coding program (https://solomon.andraspeter.com/, downloaded on 30 September 2022). Four behaviors were coded and the time (expressed as % of 6 min) and frequency of each behavior was used for further analysis.

We defined the four distinct behaviors as follows:

- Struggling (also called climbing), when the animal is almost in a vertical position, uses hard strokes and the front paws reach above the water line;
- Swimming, when the animal is in a more horizontal position and does not reach above the water with front paws as it swims around;
- Immobility (or floating), when the animal is almost still and only uses little movements to keep its head above the water;
- Diving, when the animal is fully immersed, and usually it dives to the bottom of the cylinder.

To clearly distinguish between stress-sensitive and resistant animals, we divided the trained group into three parts and considered the 1/3 group that floated the most as passive coping (P), while the group that floated the least was considered active coping (A) [33]. The middle 1/3 gray zone was not considered. The significant difference between the A and P groups supported our choice (Figure 1).

### 4.3. Quantitative PCR

**Figure 7:**
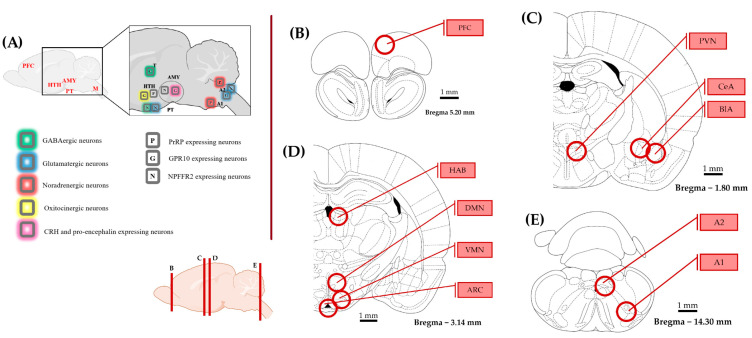
Representation of the brain areas relevant to this study. The schematics were made according to the rat brain atlas [70]. (**A**) Distribution of prolactin-releasing peptide (PrRP) and its receptors with a focus on brain areas implicated in stress coping. (**B**–**E**) The examined brain regions (circles) are illustrated on coronal sections from rostral to caudal. The circles represent the location and size of the punched tissue samples. The rostro-caudal position of the coronal sections is shown at the bottom. Abbreviations: A1, A2—noradrenergic cell groups in the medulla oblongata, AMY—amygdala, ARC—arcuate nucleus, BLA—basolateral amygdala, CeA—central amygdala, CRH—corticotropin releasing hormone, DMN—dorsomedial nucleus of the hypothalamus, G—GPR10 expressing neuron, GABA—gamma-amino-butyric acid, HAB—habenula, HTH—hypothalamus, M—medulla oblongata, N—Neuropeptide FF receptor 2 expression, P—PrRP, PT—pituitary, PFC—prefrontal cortex, PVN—paraventricular nucleus of the hypothalamus, T—thalamus, and VMN—ventromedial nucleus of the hypothalamus.

To collect the samples from 10 brain areas, the brain was manually cut into 1 mm slices, and a tissue cylinder with a diameter of 1 mm was cut out with a tissue sampler over dry ice according to the Paxinos & Watson brain atlas [70] (Figure 7). All equipment was cleaned with 70% alcohol between animals. Samples were placed directly into the first buffer solution of the RNA isolation kit (RNeasy Mini Kit, Quiagen, Venlo, The Netherlands cat.no.: 74104). Total RNA isolation was carried out following the manufacturer’s protocol. RNA samples were stored at −80 °C. A High-Capacity RNA-to-DNA kit (by Thermo Fischer Scientific, Braunschweig, Germany, cat. no.: 4387406) was used for RT-PCR with 1 µg of isolated RNA, 1 µL of enzyme mix and 10 µL of buffer solution for each sample. The cDNA solution was diluted 2.5×. Primers designed with BLAST (https://www.ncbi.nlm.nih.gov/tools/primer-blast/ accessed on 17 November 2022) and manufactured by Integrated DNA Technologies (see sequences below) were validated in silico (with BLAST) and used in a 10× dilution. *Gapdh* (glyceraldehyde 3-phosphate dehydrogenase) was used as reference gene (Table 3). The qPCR master mix was prepared with 10 µL of SYBR GREEN (Meridian, cat.no.: BIO-94020), 1-1 µL of diluted primers, 3 µL of RNase-free water, and 3 µL of cDNA sample. Every new batch of master mix was tested as no-template controls, and fluorescence did not reach the detection threshold. Samples were run in duplicates on 96-well plates with the Applied Biosystems QuantStudio 3 Real-Time PCR System. The qPCR setup is disclosed in Supplementary Table S5. For data analysis, the ΔΔCT method was used [71].

### 4.4. RNAscope In Situ Hybridization Combined with Immunostaining

#### 4.4.1. Sample Collection and Preparation

Brains from the transcardially perfused animals were collected and subjected to 2 × 24 h post fixation in 4% PFA solution. For cryoprotection, they were immersed in 30% sucrose solution for another 2 × 24 h at 4 °C. While frozen on dry ice, 30 µm thick coronal slices were cut with a manually operated sliding microtome (Leica SM2010R, Leica Biosystems, Deer Park, TX, USA). Sections were stored in antifreeze solution at −20 °C.

#### 4.4.2. RNAscope and IHC

The RNAscope was carried out as described previously [72,73]. Briefly, an endogenous peroxidase-blocking pretreatment was applied with 1% hydrogen–peroxide for 30 min in free-floating, then the slices were mounted on SuperFrost Ultra Plus adhesion slides (Thermo Fischer Scientific, Braunschweig, Germany, cat.no.: 10417002). The slides were baked at 60 °C for 1 h, then were postfixed with 4% PFA both before and after a proteinase-K treatment. Rat-specific probes *Prrp* (Bio-Techne, Minneapolis, MN, USA, cat. no.: 518741), *Crh* (cat. no.: 318931-C3), *Vglut2* (cat. no.: 317011-C2), and *Gad1* (cat. no.: 316401-C2) were hybridized with the samples. The assay (signal amplification and sequential channel development) was completed with RNAscope Multiplex Fluorescent Reagent Kit v.2 (Advanced Cell Diagnostics, Newark, CA, USA) according to the manufacturer’s protocol. 4′,6-diamidino-2-phenylindole (DAPI, cat. no.: 323108, Advanced Cell Diagnostics, Newark, CA, USA) was used to detect cell nuclei. Sections were cover-slipped with ProLong Antifade Mountant (Thermo Fischer Scientific) for confocal microscopy.

The sections with combined immunostaining were washed in PBS for 2 × 15 min after the channel development was complete and incubated overnight with polyclonal rabbit anti-PrRP31 (Phoenix Pharmaceuticals, Bulingame, CA, USA, cat. no.: H-008-52; diluted 1:2000 with 2% normal goat serum) or anti-TH (Abcam, Cambridge, UK, cat. no.: ab112; diluted 1:2000 with 2% normal goat serum). Then, sections were washed for 2 × 15 min in PBS and treated in Cyanine3-conjugated donkey anti-rabbit (Jackson Immunoresearch, St Thomas’ Place, UK, cat. no.: 711–165-152) or Alexa Fluor 647-conjugated donkey anti-rabbit (Jackson Immunoresearch, cat. no.: 711–605-152) secondary antibody, respectively, diluted 1:500 in PBS with 2% normal goat serum for 3 h at RT. After rinses, cell nuclei were stained with DAPI, and sections were covered with the mentioned mountant.

To obtain fluorescent images, an Olympus Fluoview FV-1000 laser scanning confocal microscope and FluoView FV-1000S-IX81 image acquisition software system (Olympus, Tokyo, Japan) were used. The confocal aperture was set to 80 µm. We conducted the analog sequential scanning with a 60 × objective lens (NA: 1.49 oil). We applied an optical thickness of 1 µm, and the resolution was set to 1024 × 1024 pixels. The excitation time was set to 4 µs per pixel. The following virtual colors were used for the fluorescent signals: blue (DAPI), red (Cy3), green (Fluorescein), and white (Cy5 and Alexa 647).

### 4.5. Statistics and Software

Raw data were organized in Microsoft Excel and outliers (outside 3 standard deviations) were winsorized. Preparing graphs as well as one-way ANOVA, followed by Tukey’s multiple comparisons test, were performed using GraphPad Prism version 10.4.1 for Windows, GraphPad Software, Boston, MA, USA. Figures were prepared in Microsoft Power Point with the help of BioRender online application and flaticon.com (both accessed on 25 April 2025). Data are represented as mean ± SEM.

## Figures and Tables

**Table 1:** Summary of qPCR data.

		*Prrp*			*Gpr10*			*Npff2*		
		CTR	A	P	CTR	A	P	CTR	A	P
A1	Mean	0.970	1.490	0.590	0.973	0.843	0.755	0.940	0.844	0.646
	SEM	0.211	0.201	0.115	0.204	0.122	0.086	0.132	0.127	0.044
	STATt	F(2, 20) = 6.705, *p* = 0.006			F(2, 20) = 0.596, *p* = 0.560			F(2, 20) = 0.970, *p* = 0.166		
A2	Mean	0.891	0.642	0.528	0.952	0.737	0.711	0.931	0.619	0.535
	SEM	0.280	0.070	0.090	0.200	0.046	0.053	0.248	0.105	0.048
	STATt	F(2, 20) = 1.266, *p* = 0.304			F(2, 20) = 1.311, *p* = 0.292			F(2, 20) = 1.926, *p* = 0.172		
VMN	Mean				0.934	1.115	0.690	0.925	0.858	0.633
	SEM				0.163	0.174	0.074	0.141	0.135	0.046
	STATt				F(2, 20) = 2.411, *p* = 0.116			F(2, 20) = 1.814, *p* = 0.189		
DMN	Mean	0.885	0.684	1.020	1.005	1.231	0.939	0.896	0.742	0.598
	SEM	0.192	0.056	0.205	0.159	0.189	0.115	0.170	0.032	0.096
	STATt	F(2, 20) = 1.117, *p* = 0.347			F(2, 20) = 0.978, *p* = 0.393			F(2, 20) = 1.863, *p* = 0.181		
PVN	Mean				0.994	1.033	1.215	0.895	0.949	0.902
	SEM				0.127	0.126	0.195	0.173	0.151	0.115
	STATt				F(2, 20) = 0.580, *p* = 0.569			F(2, 20) = 0.042, *p* = 0.959		
ARC	Mean				0.913	1.543	0.721	0.902	0.720	0.459
	SEM				0.180	0.273	0.096	0.168	0.077	0.034
	STATt				F(2, 20) = 4.856, *p* = 0.019			F(2, 20) = 4.736, *p* = 0.021		
CEA	Mean				1.010	0.775	0.787	0.957	0.698	0.585
	SEM				0.123	0.094	0.111	0.190	0.088	0.055
	STATt				F(2, 20) = 1.406, *p* = 0.268			F(2, 20) = 2.529, *p* = 0.105		
BLA	Mean				0.935	0.842	1.119	0.860	0.626	0.634
	SEM				0.157	0.110	0.191	0.210	0.105	0.090
	STATt				F(2, 20) = 0.840, *p* = 0.446			F(2, 20) = 0.880, *p* = 0.430		
HAB	Mean				0.998	0.802	0.677	0.939	0.735	0.405
	SEM				0.160	0.074	0.135	0.179	0.122	0.046
	STATt				F(2, 19) = 1.586, *p* = 0.231			F(2, 19) = 5.001, *p* = 0.018		
PFC	Mean				0.989	1.015	1.128	0.909	0.892	1.201
	SEM				0.102	0.181	0.085	0.178	0.186	0.152
	STATt				F(2, 20) = 0.313, *p* = 0.735			F(2, 20) = 1.039, *p* = 0.372		

Relative fold change means by group, results of one-way ANOVA, significant differences highlighted in yellow. n = 7–8/group. mRNA abbreviations: Prrp—prolactin-releasing peptide, Gpr10—specific receptor to PrRP, and Npff2—neuropeptide FF receptor 2; groups: CTR—control, A—active coping, and P—passive coping; brain regions: A1 and A2—noradrenergic nuclei of the medulla, VMN—ventromedial nucleus of the hypothalamus, DMN—dorsomedial nucleus of the hypothalamus, PVN—paraventricular nucleus of the hypothalamus, ARC—arcuate nucleus, CEA—central amygdala, BLA—basolateral amygdala, HAB—habenula, and PFC—prefrontal cortex.

**Table 2:** Excerpt from Supplementary Table S4: threshold cycle values in habenula (HAB) and the prefrontal cortex (PFC).

		Control			Active Coping			Passive Coping		
		*Gapdh*	*Gpr10*	*Npffr2*	*Gapdh*	*Gpr10*	*Npffr2*	*Gapdh*	*Gpr10*	*Npffr2*
HAB	Mean	18.753	27.511	27.234	19.732	28.802	28.464	18.282	27.968	27.578
	SEM	0.333421	0.19323	0.239264	1.860402	1.077033	1.048046	0.135559	0.193177	0.338596
	n	7	7	7	7	8	7	8	8	8
PFC	Mean	18.769	28.650	28.275	18.666	28.872	28.363	18.973	28.542	28.249
	SEM	0.458155	0.215656	0.251408	0.346269	0.300799	0.296985	0.29918	0.507652	0.444487
	n	7	7	7	8	8	8	8	8	8

Abbreviations: Gapdh: glyceraldehyde-3-phosphate dehydrogenase; Prrp: prolactin-releasing peptide; Gpr10: specific PrRP receptor; Npffr2: non-specific PrRP receptor.

**Table 3:** Primer sequences.

*Gapdh*	forward	5-AAA AGG GTC ATC TCC GC-3
	reverse	5-GCC ATC CAC AGT CTT CTG AG-3
*Prrp*	forward	5-CCC CTG ATA TCA ATC CTG CC-3
	reverse	5-CCA CGC TGA GAG ACC TTG G-3
*Gpr10*	forward	5-AGG CTT CAG AGA GCA ATG TG-3
	reverse	5-ACA TGA GCA CAT CGG ACA AG-3
*Npff2*	forward	5-GAC CCC ATC TGC AAT CAT GT-3
	reverse	5-AGA TAG TGG CAA AGA GCA CG

Gapdh—glyceraldehyde 3-phosphate dehydrogenase, Prrp—prolactin-releasing peptide, Gpr10—specific receptor to PrRP, and Npff2—neuropeptide FF receptor 2.

## Data Availability

The data are available in the Supplementary Material.

## Supplementary Materials

The following supporting information can be downloaded at: https://www.mdpi.com/article/10.3390/ijms26094155/s1.
