# Supplementary material for: Prolactin-Releasing Peptide System as a Potential Mechanism of Stress Coping: Studies in Male Rats
Source: Int J Mol Sci. 2025 Apr 27;26(9):4155. doi: 10.3390/ijms26094155 (PMC12071775; doi:10.3390/ijms26094155)
Supplement: Supplementary file 1 [file ijms-26-04155-s001.zip › ijms-3566895-supplementary.pdf]

Supplementary Table S1. Diagnostic criteria for major depression disorder in adults according to DSM 5TR [1] and their applicability to animal models

		Diagnostic criteria of major depression for adults		*Interpreting options to animal models*	*Ref.*
A	**1**	Depressed mood	and/or	Forced swim test<br>Tail suspension test	[2-4]
	**2**	Diminished interest/pleasure		Sucrose preference test<br>Splash test	[5-8]
	**3**	More than 5% unintentional weight loss		Monitoring the animal's weight	[9-11]
	**4**	Insomnia or hypersomnia		Monitoring sleep (for example with EEG)	[12, 13]
	**5**	Observable psychomotor agitation or retardation		Open field test	[7, 14]
	**6**	Fatigue, loss of energy			
	**7**	Feeling worthless, inappropriate guilt		Not applicable	-
	**8**	Difficulty in concentration, indecisiveness		Morris water maze<br>Elevated plus maze	[11, 15]
	**9**	Recurrent thoughts of death, suicidal ideation without specific plan		Not applicable	-
B		The symptoms cause clinically significant distress or impairment in social, occupational, or other important areas of functioning.		(Not applicable)	-
C		The episode is not attributable to the physiological effects of a substance or another medical condition.			
D		The episode is not related to the schizophrenia spectrum.			
E		There has never been a manic episode or a hypomanic episode.			

1. APA. *Diagnostic and Statistical Manual of Mental Disorders, Fifth Edition, Text Revision (Dsm-5-Tr)*. 5th ed., text rev. ed, Diagnostic and Statistical Manual of Mental Disorders, 2022.
2. Porsolt, R. D., G. Anton, N. Blavet, and M. Jalfre. "Behavioural Despair in Rats: A New Model Sensitive to Antidepressant Treatments." *Eur J Pharmacol* 47, no. 4 (1978): 379-91.
3. Porsolt, R. D., A. Bertin, and M. Jalfre. "Behavioral Despair in Mice: A Primary Screening Test for Antidepressants." *Arch Int Pharmacodyn Ther* 229, no. 2 (1977): 327-36.
4. Castagne, V., P. Moser, S. Roux, and R. D. Porsolt. "Rodent Models of Depression: Forced Swim and Tail Suspension Behavioral Despair Tests in Rats and Mice." *Curr Protoc Pharmacol* Chapter 5 (2010): Unit 5 8.
5. Markov, D. D. "Sucrose Preference Test as a Measure of Anhedonic Behavior in a Chronic Unpredictable Mild Stress Model of Depression: Outstanding Issues." *Brain Sci* 12, no. 10 (2022).
6. Berrio, J. P., and O. Kalliokoski. "Rethinking Data Treatment: The Sucrose Preference Threshold for Anhedonia in Stress-Induced Rat Models of Depression." *J Neurosci Methods* 395 (2023): 109910.
7. Sampaio, O. G. M., Saar Santos, Mbmv Damasceno, L. B. Joventino, A. R. Campos, and M. B. Cavalcante. "Repeated Ovarian Hyperstimulation Promotes Depression-Like Behavior in Female Mice." *Horm Behav* 164 (2024): 105589.
8. Magalhaes, Code, R. A. L. Sousa, B. F. Mendes, I. R. Dias, R. R. S. Pereira, G. C. Pereira, K. S. Lee, M. F. D. Peixoto, and R. C. Cassilhas. "Accumulated Hiit Inhibits Anxiety and Depression,

Improves Cognitive Function, and Memory-Related Proteins in the Hippocampus of Aged Rats." *Exp Brain Res* 242, no. 8 (2024): 1871-79.

9. Dess, N. K., and C. D. Chapman. "Individual Differences in Taste, Body Weight, and Depression in the "Helplessness" Rat Model and in Humans." *Brain Res Bull* 24, no. 5 (1990): 669-76.
10. Rutkowska, M., M. Trocha, M. Szandruk, W. Slupski, and J. Rymaszewska. "Effects of Supplementation with Fish Oil and N-3 Pufas Enriched Egg Yolk Phospholipids on Anhedonic-Like Response and Body Weight in the Rat Chronic Mild Stress Model of Depression." *Pharmazie* 68, no. 8 (2013): 685-8.
11. Wu, R., Y. Xiong, Y. Gu, L. Y. Cao, S. Y. Zhang, Z. X. Song, P. Fan, and L. Lin. "Traditional Pediatric Massage Enhanced Hippocampal Gr, Bdnf and Igf-1 Expressions and Exerted an Anti-Depressant Effect in an Adolescent Rat Model of Cums-Induced Depression." *Neuroscience* 542 (2024): 47-58.
12. Baxter, M. G., M. J. Leach, A. A. Miller, D. M. Sethna, and P. L. Wheatley. "Some Behavioural and Eeg Studies on the Behavioural Depression Induced in the Rat by Ethanolamine O-Sulphate, an Inhibitor of Gaba-Transaminase [Proceedings]." *Br J Pharmacol* 57, no. 3 (1976): 431P-32P.
13. Savelyev, S. A., T. Rantamaki, K. M. Rytkonen, E. Castren, and T. Porkka-Heiskanen. "Sleep Homeostasis and Depression: Studies with the Rat Clomipramine Model of Depression." *Neuroscience* 212 (2012): 149-58.
14. Sun, Y., D. Zhao, Q. Song, T. Cong, L. Li, H. Wu, and Z. Xiao. "Nmt2 Alleviates Depression-Like Behavior in a Rat Model of Chronic Unpredictable Stress: An Integrated Proteomic and Phosphoproteomic Analysis." *J Psychiatr Res* 176 (2024): 119-28.
15. Hui, Z., M. Guang-Yu, X. Chong-Tao, Y. Quan, and X. Xiao-Hu. "Phenytoin Reverses the Chronic Stress-Induced Impairment of Memory Consolidation for Water Maze Training and Depression of Ltp in Rat Hippocampal Ca1 Region, but Does Not Affect Motor Activity." *Brain Res Cogn Brain Res* 24, no. 3 (2005): 380-5.

Supplemetary Table S2. Raw FST data. Column marked with # represents the animals' identifying number

		STRUGGLING			SWIMMING			IMMOBILITY			DIVING		
	#	%	Frequency	Duration (s)	%	Frequency	Duration (s)	%	Frequency	Duration (s)	%	Frequency	Duration (s)
**CTR**	5	42.04	5	153.2	19.32	10	70.4	36.06	10	131.4	1.43	2	5.2
	10	51.45	6	188.4	35.55	12	130.2	11.36	6	41.6			
	15	27.91	4	102	30.54	7	111.6	40.12	4	146.6			
	16	27.49	7	106.6	17.59	12	68.2	47.81	8	185.4			
	20	52.66	10	202	22.16	13	85	17.15	6	65.8	1.93	4	7.4
	25	50.68	13	194	34.9	15	133.6	8.52	4	32.6			
	31	22.09	6	82.8	37.41	13	140.2	26.2	10	98.2	10.41	8	39
**R**	4	24.62	5	89.8	35.69	7	130.2	37.66	2	137.4	0.77	1	2.8
	6	34.79	6	127	26.63	10	97.2	37.26	6	136			
	9	50.06	6	181.8	23.02	10	83.6	26.1	4	94.8			
	11	26.43	4	96.8	55.76	7	204.2	16.17	3	59.2			
	14	25.96	3	94.6	35.95	13	131	36.5	10	133	0.44	1	1.6
	18	46.28	4	172.8	11.57	5	43.2	38.62	4	144.2			
	22	42.09	6	164.4	17.41	10	68	32.72	6	127.8			
	27	21.49	4	80.4	37.94	8	140	34.36	4	126.8	3.52	3	13
**V**	2	25.52	2	92.6	3.53	3	12.8	70.23	3	254.8			
	3	22.47	4	82.6	8.98	4	33	66.05	3	242.8	0.49	1	1.8
	7	14.73	3	53.6	24.24	9	88.2	60.03	7	218.4			
	12	20.44	7	74.8	16.45	9	60.2	61.53	6	225.2			
	23	22	7	86.6	13.47	12	53	56.05	7	220.6			
	24	16.6	3	63	18.12	8	68.8	59.69	6	226.6	0.47	1	1.8
	28	11.69	2	42.6	10.87	4	39.6	73.33	3	267.2	2.96	2	10.8
	30	18.94	4	69.2	17.19	3	62.8	59.77	1	218.4	2.68	1	9.8

Abbreviations: CTR: control; R: resilient; V: vulnerable

Supplementary Table S3. Statistics on FST data

	STRUGGLING		SWIMMING		IMMOBILITY		DIVING	
	%	Frequency	%	Frequency	%	Frequency	%	Frequency
**CTR-mean**	39.19	7.29	28.21	11.71	26.75	6.86	4.59	4.67
**SEM**	4.95	1.19	3.15	0.97	5.71	0.96	2.91	1.76
**R - mean**	33.97	4.75	30.50	8.75	32.42	4.88	1.58	1.67
**SEM**	3.88	0.41	4.91	0.88	2.72	0.88	0.98	0.67
**V-mean**	19.05	4.00	14.11	6.50	63.34	4.50	1.65	1.25
**SEM**	1.60	0.71	2.25	1.21	2.10	0.80	0.68	0.25
**ANOVA F value (2, 20)**	8.313	4.448	6.069	6.165	29.51	1.995	0.7323	1.359
**ANOVA p value**	0.0024	0.0245	0.0087	0.0082	< 0.0001	0.1622	0.4933	0.2797

Abbreviations: CTR: control; R: resilient; V: vulnerable

Supplementary Table S4. CT values from raw data of qPCR.

A1		*Gapdh*		*Prrp*		*Gpr10*		*Npff2*	
		CT1	CT2	CT1	CT2	CT1	CT2	CT1	CT2
CTR	5	19.981	20.133	27.383	27.412	27.747	27.366	26.524	26.794
	10	18.231	18.264	26.271	25.996	28.129	28.004	27.176	27.226
	15	18.670	18.555	27.918	27.958	28.428	28.188	27.051	27.086
	16	18.889	18.892	26.707	26.735	27.840	27.958	26.794	26.887
	20	19.065	18.985	28.594	28.561	26.517	26.531	26.863	26.786
	25	18.565	18.486	25.224	25.253	26.978	26.758	26.943	26.963
	31	18.996	18.996	28.715	28.731	28.630	28.534	27.947	27.973
R	4	18.167	17.987	25.638	25.616	27.768	27.625	27.038	27.286
	6	19.221	19.128	26.374	26.324	26.560	26.417	25.391	25.505
	9	17.058	17.054	25.285	25.284	26.990	26.952	26.113	26.059
	11	18.535	18.563	25.797	25.793	27.162	27.292	26.897	27.172
	14	18.085	18.149	27.298	27.200	26.697	26.607	26.248	26.276
	18	18.316	18.248	25.596	25.670	27.530	27.430	27.247	27.243
	22	17.388	17.383	25.947	29.743	26.960	26.639	25.969	25.977
	27	19.130	19.078	25.872	25.794	28.684	28.598	26.961	26.942
V	2	19.509	19.389	26.233	26.265	28.863	28.843	27.801	27.997
	3	18.984	18.985	27.888	27.986	27.757	27.941	27.728	27.488
	7	17.045	17.007	26.109	26.033	25.710	25.874	24.681	24.723
	12	20.978	20.889	29.081	28.919	29.122	29.133	28.457	28.588
	23	17.365	17.308	27.185	27.136	26.112	26.128	26.339	26.425
	24	19.285	19.274	28.621	28.854	29.959	30.014	29.633	29.629
	28	18.336	18.455	27.739	27.749	27.027	27.223	27.152	27.327
	30	19.501	19.045	28.916	29.088	28.859	29.391	28.213	28.077

A2		*Gapdh*		*Prrp*		*Gpr10*		*Npff2*	
		CT1	CT2	CT1	CT2	CT1	CT2	CT1	CT2
CTR	5	20.827	20.746	28.159	28.236	28.364	28.147	27.206	27.337
	10	18.000	18.255	26.219	26.115	27.268	27.322	26.804	26.816
	15	18.512	18.440	27.199	27.160	27.628	27.607	26.578	26.538
	16	18.232	18.231	23.415	23.461	26.207	26.218	26.187	26.277
	20	24.133	19.316	28.384	28.532	27.794	27.493	26.926	27.214
	25	17.438	17.455	22.349	22.447	25.880	25.856	26.129	26.108
	31	17.841	17.736	25.807	25.706	25.639	25.767	24.976	25.069
R	4	18.905	18.802	27.132	27.219	27.322	27.247	27.296	27.296
	6	20.922	20.829	27.529	27.469	28.469	28.150	27.167	27.118
	9	17.075	16.978	23.513	23.416	24.550	24.451	25.848	25.959
	11	18.301	18.274	25.796	25.671	27.086	27.036	26.974	27.000
	14	18.610	18.590	26.992	26.807	27.458	26.992	26.253	26.379
	18	18.110	18.204	25.108	25.237	26.646	26.667	27.009	27.246
	22	18.469	18.492	25.317	25.241	26.479	26.486	26.722	26.797
	27	20.970	20.799	28.867	28.790	29.329	29.238	28.054	28.120

**V**	2	20.179	20.044	26.988	27.063	28.283	28.018	28.239	28.079
	3	17.078	16.705	24.771	24.739	25.275	25.373	25.139	24.735
	7	17.356	17.169	26.287	26.184	25.905	25.973	24.814	25.207
	12	22.444	22.350	29.502	29.527	29.531	29.619	28.991	29.207
	23	16.965	16.884	21.848	21.818	24.038	24.034	26.495	26.497
	24	18.966	18.940	27.259	27.180	27.104	27.105	26.986	26.792
	28	18.766	18.555	25.629	25.760	27.447	27.394	26.940	26.967
	30	19.463	19.036	27.848	27.813	28.311	28.402	27.937	28.097
**VMN**		***Gapdh***		***Prrp***		***Gpr10***		***Npff2***	
		CT1	CT2	CT1	CT2	CT1	CT2	CT1	CT2
**CTR**	5	21.001	20.910	28.136	28.193	28.113	28.071	27.324	27.176
	10	18.479	18.440	28.569	28.558	26.392	26.420	27.128	27.228
	15	16.593	16.576	26.290	26.282	25.607	25.735	25.588	25.513
	16	17.891	17.865	26.887	26.873	25.453	25.487	25.786	25.848
	20	18.357	18.354	28.033	27.958	25.876	25.848	26.428	28.147
	25	19.285	19.189	28.328	28.351	25.696	25.720	27.446	27.677
	31	17.605	17.659	27.324	27.293	26.310	26.252	26.128	26.361
**R**	4	18.227	18.493	29.848	29.739	26.207	26.030	28.192	27.959
	6	19.382	19.206	26.737	26.898	26.739	27.176	25.955	26.005
	9	17.217	17.183	25.522	25.458	25.580	25.544	25.885	25.763
	11	19.907	19.356	29.520	28.940	25.765	25.687	27.674	27.712
	14	17.572	17.531	26.818	26.780	25.182	25.232	25.993	25.973
	18	16.710	16.675	26.978	26.894	23.590	23.515	25.363	25.413
	22	17.797	17.731	28.455	28.326	25.335	25.469	26.796	26.910
	27	17.628	17.746	27.923	27.977	0.000	0.000	26.053	25.880
**V**	2	18.912	18.692	28.023	27.999	27.161	27.176	27.576	27.693
	3	19.952	19.856	29.962	29.663	29.266	29.207	28.966	28.969
	7	17.998	18.111	29.547	29.737	25.311	25.374	26.900	26.994
	12	19.116	19.022	28.596	28.463	26.590	26.582	27.601	27.646
	23	16.515	16.445	24.680	24.640	25.093	25.187	26.110	26.081
	24	18.721	18.726	25.075	25.026	27.445	27.496	27.190	27.140
	28	18.070	18.001	26.248	26.271	26.138	26.161	27.157	27.117
	30	16.892	16.731	25.838	25.541	25.097	25.170	26.320	26.644
**DMN**		***Gapdh***		***Prrp***		***Gpr10***		***Npff2***	
		CT1	CT2	CT1	CT2	CT1	CT2	CT1	CT2
**CTR**	5	20.534	20.453	28.016	28.066	27.721	27.780	27.005	26.903
	10	20.979	20.990	30.075	29.972	29.081	29.163	29.043	29.183
	15	16.748	17.132	27.016	26.963	26.908	26.828	25.637	25.652
	16	17.853	17.806	27.449	27.403	26.854	26.694	26.325	26.275
	20	18.311	18.238	28.733	28.665	26.258	26.064	26.707	26.636
	25	17.890	17.846	27.614	27.629	25.243	25.191	26.627	26.723
	31	17.944	17.931	27.669	27.517	26.436	26.421	26.550	26.634

R	4	20.051	19.941	30.518	30.703	26.970	26.890	29.367	29.647
	6	20.729	20.737	29.227	29.223	28.565	28.418	28.308	28.193
	9	16.900	16.825	25.393	25.389	25.986	25.983	25.672	25.721
	11	18.412	18.457	28.252	28.305	25.408	25.429	26.846	26.867
	14	17.893	17.857	27.785	27.754	25.889	25.882	26.456	26.534
	18	17.103	17.105	26.752	26.624	24.038	23.954	25.018	25.100
	22	19.032	19.075	29.845	27.216	26.795	26.821	27.445	27.536
	27	17.641	17.832	28.236	28.306	27.061	27.660	25.971	26.077
V	2	19.401	19.320	28.671	28.665	26.656	26.556	28.599	28.298
	3	20.330	20.131	29.782	29.688	29.384	29.402	28.765	29.145
	7	17.201	17.250	28.335	28.489	25.760	25.672	26.808	26.784
	12	21.796	21.909	28.429	28.376	28.518	28.583	28.140	28.143
	23	16.618	16.532	27.192	27.327	26.101	25.991	25.816	25.758
	24	18.745	18.793	27.417	27.415	27.450	27.391	26.878	26.906
	28	19.127	19.146	27.790	27.752	26.898	26.974	28.024	28.025
	30	18.140	18.303	28.571	28.400	27.011	26.954	27.713	27.662
**PVN**		*Gapdh*		*Prrp*		*Gpr10*		*Npff2*	
		CT1	CT2	CT1	CT2	CT1	CT2	CT1	CT2
**CTR**	5	19.624	19.500	26.937	27.045	27.125	27.068	26.138	26.217
	10	17.985	18.010	28.094	28.013	26.896	26.983	26.748	26.737
	15	19.014	18.974	28.707	28.546	27.441	27.492	27.513	27.398
	16	17.890	17.886	26.854	26.876	26.083	26.137	25.836	25.866
	20	18.280	18.377	28.587	28.482	27.179	27.265	27.025	27.300
	25	17.704	17.719	28.435	28.300	27.037	27.265	27.557	27.602
	31	18.269	18.258	27.852	27.692	26.095	26.027	26.897	26.969
**R**	4	17.647	17.640	29.991	30.251	27.039	26.835	27.571	27.252
	6	20.488	20.440	27.891	27.719	28.058	27.993	26.728	26.967
	9	17.342	17.309	25.885	25.711	25.766	25.849	26.291	26.430
	11	19.315	19.342	28.221	28.169	27.970	27.956	27.188	27.279
	14	18.542	18.467	28.993	28.887	27.347	27.505	27.251	27.576
	18	18.515	18.425	28.076	28.052	27.090	26.992	26.022	26.058
	22	17.504	17.603	27.826	27.750	25.695	25.860	26.504	26.441
	27	18.536	18.615	28.319	28.365	25.438	25.358	27.165	26.635
**V**	2	18.903	18.978	27.977	27.883	25.794	25.907	27.585	27.627
	3	20.086	19.972	28.196	28.204	27.793	27.858	27.466	27.675
	7	19.120	19.216	28.566	28.415	27.425	27.426	26.799	27.017
	12	18.946	18.839	27.961	27.947	26.579	26.580	26.880	26.820
	23	17.575	17.444	28.110	27.988	27.179	27.212	26.342	26.514
	24	17.851	17.799	24.943	24.901	27.237	27.227	26.515	26.484
	28	18.105	18.165	28.419	28.312	26.231	26.205	27.002	27.052
	30	17.046	17.106	28.383	28.121	24.798	24.942	27.131	26.830
**ARC**		*Gapdh*		*Prrp*		*Gpr10*		*Npff2*	

		CT1	CT2	CT1	CT2	CT1	CT2	CT1	CT2
**CTR**	5	19.948	20.047	27.121	27.168	26.988	26.915	26.327	26.257
	10	19.159	19.185	28.945	28.980	27.847	27.742	27.982	28.183
	15	19.333	19.280	28.299	28.141	27.625	27.648	27.615	27.814
	16	19.453	19.418	28.058	28.131	25.032	25.068	26.908	26.979
	20	20.756	20.781	30.325	30.135	28.994	29.153	29.065	29.036
	25	19.694	19.579	29.020	29.071	27.082	27.086	27.730	27.778
	31	17.687	17.609	25.395	25.329	25.684	25.604	25.868	25.873
**R**	4	18.216	18.326	28.656	28.590	24.396	24.400	27.007	27.097
	6	18.707	18.637	25.894	25.905	25.891	25.929	25.020	25.000
	9	18.875	18.834	27.049	27.050	27.575	27.690	27.479	27.563
	11	19.510	19.463	28.363	28.280	25.594	25.583	27.405	27.441
	14	18.563	18.598	28.289	28.267	25.947	26.007	27.150	27.250
	18	18.689	18.590	27.965	27.937	25.050	25.001	26.486	26.394
	22	19.499	19.503	29.204	29.066	25.320	25.344	27.707	27.968
	27	21.781	21.589	30.071	29.954	29.814	29.824	28.684	28.651
**V**	2	19.398	19.293	28.752	28.541	26.273	26.227	28.329	28.449
	3	20.886	20.756	30.062	30.075	29.248	29.038	29.220	29.174
	7	18.429	18.456	28.493	28.519	25.666	25.707	26.861	26.975
	12	21.081	20.981	30.206	30.311	27.694	27.964	29.076	28.892
	23	18.245	18.185	27.289	27.175	26.326	26.289	27.349	27.592
	24	17.837	17.917	24.326	24.369	27.064	27.125	26.581	26.448
	28	17.641	17.415	26.735	26.711	25.957	25.989	26.171	26.162
	30	18.847	18.729	28.770	28.679	27.025	27.007	28.091	28.288

**CEA**		***Gapdh***		***Prrp***		***Gpr10***		***Npff2***	
		CT1	CT2	CT1	CT2	CT1	CT2	CT1	CT2
**CTR**	5	20.825	20.801	28.000	27.808	28.121	28.204	27.049	26.973
	10	19.302	19.306	28.949	29.133	26.989	27.013	28.205	28.321
	15	18.298	18.178	26.879	26.747	26.936	26.960	26.222	26.258
	16	18.757	18.705	26.981	26.982	27.125	27.127	26.241	26.125
	20	18.648	18.679	27.297	27.313	26.463	26.572	26.351	26.172
	25	17.572	17.485	27.233	27.164	26.266	26.228	26.819	26.743
	31	18.657	18.634	29.014	29.034	28.852	28.940	28.386	27.992
**R**	4	18.767	18.839	29.462	29.362	27.714	27.921	28.575	28.604
	6	21.293	21.261	28.744	28.640	29.000	28.696	27.893	27.837
	9	18.597	18.537	26.994	27.017	27.225	27.093	27.002	27.080
	11	18.915	18.864	27.748	27.755	26.976	27.017	27.051	27.152
	14	18.602	18.569	28.376	28.313	27.844	27.855	27.181	27.043
	18	18.559	18.464	27.405	27.413	26.577	26.513	25.973	25.987
	22	16.764	16.774	26.985	26.905	25.729	25.592	26.107	25.986
	27	17.834	17.793	28.122	28.424	27.996	27.972	27.231	27.071
**V**	2	20.769	20.073	29.188	28.887	29.668	29.154	29.357	28.806
	3	20.882	20.683	29.916	29.631	28.946	29.076	28.665	28.716

	7	18.235	18.297	29.095	28.857	27.947	28.077	27.669	27.436
	12	18.740	18.738	28.238	28.104	27.904	27.987	27.154	27.055
	23	17.144	17.091	27.556	27.489	26.569	26.584	26.962	26.869
	24	17.538	17.551	25.536	25.443	22.293	22.284	26.203	26.050
	28	18.436	18.623	28.441	28.247	27.091	26.636	27.334	27.490
	30	20.715	20.478	29.859	29.644	29.001	28.839	29.252	29.253
**BLA**		***Gapdh***		***Prrp***		***Gpr10***		***Npff2***	
		CT1	CT2	CT1	CT2	CT1	CT2	CT1	CT2
**CTR**	5	20.481	20.488	27.656	27.613	27.924	27.922	26.748	26.794
	10	19.391	19.414	29.818	29.665	29.164	29.176	28.986	28.580
	15	19.055	18.978	28.740	28.233	28.321	28.092	28.323	28.294
	16	18.368	18.407	27.876	27.739	27.829	27.803	26.863	27.021
	20	19.222	19.258	28.785	28.600	27.657	27.854	27.921	27.735
	25	17.202	17.118	27.958	27.938	27.158	27.166	27.572	27.749
	31	18.289	18.191	28.186	28.202	28.295	28.135	27.400	27.536
**R**	4	18.483	18.409	28.948	28.876	27.993	28.060	28.202	28.254
	6	20.824	20.772	28.125	28.028	28.290	28.294	27.198	27.779
	9	18.221	18.131	27.220	27.179	27.598	27.789	27.425	27.580
	11	19.361	19.245	28.538	28.364	27.794	27.920	27.726	27.851
	14	19.028	18.916	29.648	29.647	29.023	29.043	28.411	28.598
	18	17.683	17.483	27.252	27.220	26.225	26.237	26.309	26.013
	22	16.995	16.989	27.710	27.768	26.330	26.275	27.179	27.285
	27	17.315	17.145	28.299	28.241	27.506	27.446	26.951	26.996
**V**	2	20.378	20.212	29.127	28.971	29.053	29.044	29.196	29.062
	3	19.278	19.134	28.406	28.412	27.191	27.987	27.508	27.544
	7	17.825	17.895	29.692	29.535	28.592	28.582	28.088	28.218
	12	18.433	18.429	27.826	27.834	26.676	26.635	26.968	27.008
	23	19.174	19.064	29.087	29.048	27.709	27.614	28.405	28.687
	24	18.034	18.031	26.977	26.981	24.908	24.903	26.347	26.301
	28	17.880	17.877	29.965	29.924	28.830	29.286	28.977	28.959
	30	20.338	20.261	29.268	29.217	29.076	28.927	28.766	28.946
**HAB**		***Gapdh***		***Prrp***		***Gpr10***		***Npff2***	
		CT1	CT2	CT1	CT2	CT1	CT2	CT1	CT2
**CTR**	5	20.575	20.458	27.593	27.468	27.670	27.732	26.641	26.722
	10	18.533	18.446	27.581	27.383	25.583	25.687	26.342	26.551
	15	18.325	18.297	27.597	27.593	26.798	26.766	27.175	27.094
	16	18.177	18.085	27.188	27.154	27.288	27.161	26.806	26.342
	20	18.779	18.842	28.652	28.522	27.489	27.619	27.479	27.625
	25	19.327	19.207	26.957	26.892	27.154	27.141	26.568	26.562
	31	17.756	17.734	27.324	27.245	27.036	27.067	26.527	26.621
**R**	4		36.542	35.538	35.823	34.446	33.753		
	6	19.711	19.587	28.183	28.007	28.276	28.012	26.640	26.563

	9	17.486	17.526	26.075	25.943	26.193	26.261	26.137	26.117
	11	18.122	17.996	27.007	26.999	26.544	26.649	26.149	26.112
	14	17.976	18.017	27.913	27.885	27.947	27.911	26.931	26.856
	18	18.589	18.537	28.992	28.811	27.070	26.865	26.987	27.234
	22	18.229	18.191	27.988	28.064	26.495	26.116	27.473	27.507
	27	17.481	17.358	27.221	26.986	26.420	26.323	25.742	25.644
**V**	2	19.074	19.019	27.837	26.673	28.623	28.588	28.102	28.495
	3	18.249	18.084	27.468	27.327	26.540	25.661	26.432	26.361
	7	17.696	17.820	28.532	28.467	26.276	26.237	26.907	26.888
	12	18.115	18.147	27.993	27.987	25.905	25.911	26.920	27.004
	23	18.169	18.180	28.315	28.177	27.897	27.945	27.758	27.598
	24	18.489	18.399	28.549	28.557	28.736	28.912	28.765	28.557
	28	18.341	18.163	27.643	28.027	26.901	26.985	27.002	27.198
	30	18.459	18.612	29.108	28.699	28.226	28.193	28.310	28.433
**PFC**		***Gapdh***		***Prrp***		***Gpr10***		***Npff2***	
		CT1	CT2	CT1	CT2	CT1	CT2	CT1	CT2
**CTR**	5	21.474	21.415	29.318	29.016	29.101	29.460	28.348	28.294
	10	18.226	18.172	28.501	28.462	27.256	27.243	27.706	27.926
	15	18.401	18.371	28.884	28.691	27.668	27.725	28.041	28.147
	16	18.698	18.697	29.362	29.399	27.926	28.145	28.497	28.288
	20	18.482	18.420	27.800	27.808	27.478	27.472	26.853	26.896
	25	18.862	18.695	28.002	27.979	28.129	28.041	27.962	27.802
	31	17.441	17.409	28.916	28.963	27.981	27.996	28.222	28.267
**R**	4	19.971	19.945	30.158	30.054	28.876	28.657	29.313	29.288
	6	19.532	19.462	27.378	27.349	27.435	27.352	26.048	26.046
	9	17.969	17.957	28.125	28.239	27.786	27.771	27.979	27.994
	11	18.812	19.181	28.932	28.991	27.489	27.544	28.319	28.198
	14	18.792	18.748	29.359	28.728	28.637	28.640	27.997	28.973
	18	18.633	18.580	29.135	29.106	27.562	27.521	27.618	27.644
	22	16.872	16.871	29.453	29.205	27.625	27.697	28.664	28.734
	27	18.491	18.601	29.167	29.086	28.331	28.373	27.797	27.738
**V**	2	18.124	18.010	25.990	26.100	26.664	26.654	26.190	26.105
	3	18.291	17.987	28.781	28.667	27.555	27.363	27.885	27.992
	7	20.111	20.040	30.625	30.664	29.294	29.268	29.294	29.114
	12	18.967	18.914	28.601	28.577	28.036	28.110	27.597	27.625
	23	19.312	19.313	28.798	28.816	28.054	28.108	28.110	28.125
	24	18.298	18.132	27.407	27.287	26.963	26.950	26.837	26.831
	28	20.162	19.963	29.683	29.592	29.217	28.761	28.431	28.890
	30	20.155	20.012	33.675	34.213	31.959	31.626	34.212	34.022

Abbreviations: CT: threshold cycle; CTR: control; R: resilient; V: vulnerable; Gapdh: glyceraldehyde-3-phosphate dehydrogenase; Prrp: prolactin releasing peptide; Gpr10: specific PrRP receptor; Npffr2: non-specific PrRP receptor; A1, A2: noradrenerd cell groups in the medulla oblongata; VMN:

ventromedial nucleus; DMN: dorsomedial nucleus; PVN: paraventricular nucleus; ARC: arcuate nucleus; CEA: central amygdala; BLA: basolateral amygdala; HAB: habenula; PFC: prefrontal cortex

Supplementary Table S5. The qPCR setup

	Inicialization	Polymerase chain reaction – 40×			Melting curve		
		Denaturation	Annealing	Extending	1	2	3
Temperature (°C)	95	95	60	72	95	60	95
Time (min:sec)	2:00	0:05	0:10	0:10	0:15	1:00	0:15
Detection				X			X

Supplementary Table 6. Characterization of prolactin-releasing peptide mRNA (*Prrp*)-positive neurons in the medullary A1 region.

	**Number of neurons**	**Number of co-localization with *Prrp***	**Ratio of *Prrp*-co-expressing cells**
***Vglut2*-positive neurons**	25	18	72%
***Crh*-positive neurons**	17	17	100%
**TH-positive neurons**	21	18	85.71%
***Gad1*-positive neurons**	87	0	0%

Abbreviations: *Vglut2*: vesicular glutamate transporter 2 mRNA; *Crh*: corticotropin-releasing hormone mRNA; *Gad1*: glutamate decarboxylase 1 mRNA; TH: tyrosine hydroxilase enzyme
